# Supplementary material for: Patterns of Intron Gain and Loss in Fungi
Source: PLoS Biol. 2004 Nov 30;2(12):e422. doi: 10.1371/journal.pbio.0020422 (PMC532390; doi:10.1371/journal.pbio.0020422)
Supplement: Table S1 — Also available at http://genes.mit.edu/NielsenEtAl/. (4.3 MB ZIP). [file pbio.0020422.st001.zip › NielsenEtAl/html/1248.html]

AN1495.1.NCU09880.1.MG10740.1.FG00757.1


```
 CLUSTAL W (1.82) Multiple Sequence Alignments - Introns Inserted


Sequence 1: NCU09880.1	85 aa
Sequence 2: FG00757.1	84 aa
Sequence 3: MG10740.1	88 aa
Sequence 4: AN1495.1	78 aa
Alignment Length: 88 aa
Number Identitical Residues: 50 aa
Alignment Score (without introns) 2309


MG10740.1 	MGPPQPELKK0YLDKKVFVQLNGSRKVVGVVRGYD~VFLNIVLEDAFEEKDGGEKVKIGT
NCU09880.1	MAPAQPELKK0YLDKRLFVQLNGSRKVIGVLRGYD0VFLNIVLDDAVEEKDGGEKVKLGM
FG00757.1 	MAPAQPELKK0YLDKRLFVQLNGSRKVIGVLRGYD0VFLNIVLDEAVEEKDNGEKVRIGM
AN1495.1  	MPQAQPELKK0YMEKRVFCELNGNRKVIGVLRGYD0VFMNIVLDEAFEEKPGGEKVAIGM
          	*  .****** *::*::* :***.***:**:**** **:****::*.*** .**** :* 

MG10740.1 	V0VIRGNSVVMLE~AMERIGGDDRHGGGGGGR
NCU09880.1	V0TIRGNSVVMME~ALERIGGDERGGQRG---
FG00757.1 	V0VIRGNSVVMLE~ALERIGGDDRRGDR----
AN1495.1  	I0VIRGNSVVMLE0ALERIS--EK--------
          	: .********:* *:***.  ::
```
